# Impact of Bee Venom Enzymes on Diseases and Immune Responses

**DOI:** 10.3390/molecules22010025

**Published:** 2016-12-27

**Authors:** Md. Sakib Hossen, Ummay Mahfuza Shapla, Siew Hua Gan, Md. Ibrahim Khalil

**Affiliations:** 1 Laboratory of Preventive and Integrative Biomedicine, Department of Biochemistry and Molecular Biology, Jahangirnagar University, Savar, Dhaka 1342, Bangladesh; sakibhossen00@gmail.com (M.S.H.); shapla.ar@gmail.com (U.M.S.); 2 Human Genome Centre, School of Medical Sciences, Universiti Sains Malaysia, Kubang Kerian, Kelantan 16150, Malaysia

**Keywords:** bee venom, enzymes, protein, toxin, pharmacotherapeutics, *Apis mellifera*

## Abstract

Bee venom (BV) is used to treat many diseases and exhibits anti-inflammatory, anti-bacterial, antimutagenic, radioprotective, anti-nociceptive immunity promoting, hepatocyte protective and anti-cancer activity. According to the literature, BV contains several enzymes, including phospholipase A2 (PLA2), phospholipase B, hyaluronidase, acid phosphatase and α-glucosidase. Recent studies have also reported the detection of different classes of enzymes in BV, including esterases, proteases and peptidases, protease inhibitors and other important enzymes involved in carbohydrate metabolism. Nevertheless, the physiochemical properties and functions of each enzyme class and their mechanisms remain unclear. Various pharmacotherapeutic effects of some of the BV enzymes have been reported in several studies. At present, ongoing research aims to characterize each enzyme and elucidate their specific biological roles. This review gathers all the current knowledge on BV enzymes and their specific mechanisms in regulating various immune responses and physiological changes to provide a basis for future therapies for various diseases.

## 1. Introduction

Bee venom (BV) is a secretion produced by the sting apparatus of bees. Its biological purpose is to protect the bees from their enemies. Because of its anti-inflammatory, anti-bacterial, antimutagenic, radioprotective, anti-nociceptive immunity-promoting, hepatocyte-protective and anti-cancer characteristics [1,2,3,4,5,6,7], it has a long history of use in folk medicine to treat various diseases. Traditionally, live bees are used to sting acupuncture points for the affected area. BV is most effective when it is directly produced by a live bee during the late spring to early fall, when bees have good access to pollen sources and can produce potent venom; venom secreted during the winter is reported to be less potent. BV is a complex mixture containing pharmacologically active proteins, peptides and enzymes, but its composition varies among different types of bees. A recent study by Matthias et al. reported that shotgun liquid chromatography Fourier transform ion cyclotron resonance mass spectrometry analysis (LC-FT-ICR MS) detected a total of 102 proteins and peptides in *Apis mellifera* BV [8].

According to the literature [9,10], there are a variety of enzymes present in BV, among which PLA2 is the most widely investigated. PLA2 accounts for up to 12% of the BV contents. It destroys cells by breaking up phospholipids (the main component of the cell membrane), while phospholipase B breaks down blood cells. Therefore, hyaluronidase (3%) acts as a spreading factor by breaking down hyaluronic acid, a polysaccharide that is present in the interstitial fluid of connective tissue and acid phosphatase is a potent trigger of histamine release from sensitized human basophils, and α-glucosidase is involved in honey production [10]. Recent studies have also reported the presence of different classes of enzymes in BV, including esterases, proteases and peptidases, protease inhibitors and other important enzymes involved in carbohydrate metabolism.

Although several studies have reported various pharmacotherapeutic properties for some of these enzymes, the function and mechanism of each enzyme remains unclear. A growing number of reports have shown that PLA2 has protective effects against a wide range of diseases, including arthritis, asthma, Parkinson’s disease and drug-induced organ inflammation [11,12,13,14]. In comparison, the biological roles and physiochemical properties of other BV enzymes have been less well studied. In this review, we highlight different types of enzymes and summarize the latest information on BV enzymes and their biological roles and mechanisms.

## 2. Methodology

To gather fundamental information on BV enzymes, we searched The Comprehensive Enzyme Information System “BRENDA” using individual enzyme commission (EC) numbers in which all records were specific for *Apis mellifera*. For proteomic data on BV enzymes, the UniProtKB database was browsed, and only the most recently investigated data on BV enzymes were reviewed by Swiss-Prot. For updates on the domain status and graphic presentations, “Pfam version 30”, which is based on the UniProt database, was considered. Finally, to retrieve literature pertaining to the mode of actions of BV enzymes against various immune responses and the precise physiological changes involved, the MeSH Database, the vocabulary source of PubMed, was systematically explored.

## 3. Bee Venom Enzymes

Honey BV comprises 88% water. The remaining 12% includes enzymes, peptides, phospholipids, biogenic amines, amino acids, sugars, volatiles and minerals. A previous study reported that 55 enzymes are present in BV, the venom sac, sacless whole body extract and whole body commercial extracts [15]. BV contains five main types of enzymes: PLA2, phospholipase B, hyaluronidase, acid phosphatase and α-glucosidase. Phospholipase B is present in very low concentrations (1% of the dry weight of BV) in some venoms and shows a combination of PLA1 and PLA2 activity [16]. PLA2 is the most abundant enzyme found in BV; it accounts for 10%–12% of the dry weight of BV and consists of hyarulonidase (1%–2%), phosphatase (1%) and α-glucosidase (0.6%) [10]. Other classes of enzymes found in BV and reported in several studies are esterases (such as PLA2-1, PLA2-2, group XV PLA2, acid phosphatase 1, acid phosphatase 2, acid phosphatase 3, 5′-nucleotidase, carboxylesterase), proteases and peptidases (such as CLIP serine protease, CUB (complement C1r/C1 s-Uegf-Bmp1) serine protease 1, CUB serine protease 2, putative trypsin, serine protease snake precursor, dipeptidyl peptidase IV, serine carboxypeptidase, prolylcarboxypeptidase, and metalloprotease), protease inhibitors (such as Api m 6, serpin 1, serpin 2, serpin 3) and some enzymes involved in carbohydrate metabolism (such as *N*-sulfoglucosamine sulfohydrolase, and endochitinase) [8]. The identification methods and investigated biological roles of major BV enzymes are summarized in Table 1, and the details of the investigated *Apis mellifera* bee enzymes’ (including PLA2, hyaluronidase, acid phosphatase and α-glucosidase) nomenclature, sources, BV-based information, enzymatics, genomics and proteomics data are provided in Table 2.

### 3.1. Phospholipase A2

Phospholipases are enzymes that hydrolyze phospholipids into fatty acids and other lipophilic substances. They are distinguished by the type of reactions they catalyze and are mainly divided into four major classes (A, B, C and D). There are two types of PLA—PLA1 and PLA2, meanwhile, PLA2 can be subdivided into 16 groups based on structure homology, source, and localization [26]. PLA2 from BV belongs to the group III secretory PLA2 (sPLA2), which can cause allergic reactions in humans. sPLA2 mainly shows its inflammatory effects by inducing the biosynthesis of prostaglandin and other inflammatory mediators including archidonic and lysophosphatidic acids by recognizing and catalytically hydrolyzing the sn-2 acyl bond of phospholipids. It has four to seven disulfide bonds, responsible for their stability and also their folding mechanism [27]. On the other hand, calcium ion is essential for their activities. In its active state, the α-amino group is involved in a conserved hydrogen-bonding network linking the N-terminal region to the active site in which, His and Asp, the side chains of the two conserved residues, participate in the catalytic network [28]. By following a seasonal pattern, BV sPLA2 is mainly secreted into the venom, in which the highest release normally occur during the winter; it is also observed that the variation synchronized with that of the production of melittin [29].

### 3.2. Phospholipase B

This enzyme is found in low concentrations in some BV and in some snake venom. It exhibits a combination of PLA1 and PLA2 activities. Its optimum pH is between 8.5 and 10. It is most stable at 100 °C at pH 7. It can be activated in the presence of calcium and magnesium ions [16]. It acts on lyso compounds and breaks them into glycerophosphorylcholine and fatty acids [30].

### 3.3. Hyaluronidase

Family 56 encompasses a group of hyaluronidases that includes BV hyaluronidase and mammalian sperm surface proteins (PH-20) [31]. It is an endo-*N*-acetyl-d-hexosaminidase that causes the β-1,4-glycosidic bond between GlcNAc (*N*-acetyl glucosamine) and GlcA (glucosamine) in the hyaluronic acid chain to cleave into non-viscous fragments. It is secreted by the venom gland and has a molecular weight of approximately 43 kDa. The complete amino acid sequence of BV hyaluronidase contains 382 amino acids (previously reported as 349) and includes four cysteines and a number of potential glycosylation sites (UniProtKB—Q08169). It also causes an allergic reaction in humans.

### 3.4. Acid Phosphatase

Venom acid phosphatase is a glycoprotein and a potent allergen in the BV of *Apis mellifera*. It contains four possible sites of glycosylation with the sequence Asn-Xaa-Ser/Thr. The motif RHGXRSP characterizes its acid phosphatase activity and distinguishes it from the acid phosphatase enzymes of other organisms. It is responsible for IgE-mediated allergic reactions in humans [23]. It causes the release of histamine from sensitized basophils related to such manifestations as causing urticaria and flare reactions. Approximately 37% of BV-allergic patients develop Api m 3 (acid phosphatase) specific IgE, which can be used in immunotherapies [23,32].

### 3.5. α-Glucosidase

α-Glucosidase acts on the α-glycosidic bond at the non-reducing terminal side of a substrate and liberates α-glucose as a product. In *Apis mellifera Lingustica*, α–glucosidase has three isozymes (I, II, III), which have different substrate specificities [25,33,34]. However, the α-glucosidase contained in honey from the hypopharyngeal gland is α-glucosidase III, as immunological methods have confirmed. The enzyme remains stable at a pH ranging from 5 to 10 (the optimum pH is 5.5) and is denatured at a pH lower than 4.5. It is stable at 40 °C, but if it is left standing at 60 °C for 15 min, it will be completely nonfunctional. Its function is to degrade sucrose in the nectar into glucose and fructose to produce honey [24].

## 4. Investigated Mechanistic Roles of BV PLA2 and Others on Several Diseases

### 4.1. Anti-Neurodegenerative Effect of PLA2

BV PLA2 has three main domains: the unique C and N-terminal domains and the middle secretory PLA2 domains. The PLA2 domain shares a high sequence of homology with the mammalian group III secretory PLA2 enzyme. Lysophosphatidylcholine is a product of mammalian group III secretory PLA2 that plays a critical role in the growth of peripheral nerve axons, synaptic signal transmission and neuron survival [35]. It is a fusogen that helps to seal the plasma membrane of damaged axons. This has been confirmed to be unequivocal, as the PLA2 inhibitor impairs this process [36]. Thus, it is plausible that BV PLA2 exerts similar effects.

Reactive human leukocyte antigen HLA-DR-expressed microglial cells and glial reactions are associated with several different neurodegenerative diseases, such as Parkinson’s disease (PD), Alzheimer’s disease (AD), and multiple sclerosis (MS). In the case of PD, microglial cells are activated in response to neuronal damage and cause extensive and perpetuated secondary damage to the dopaminergic neurons located in the substantia nigra. Activated microglial cells express different proinflammatory cytokines, such as tumor necrosis factor alpha (TNF-α), interleukin-1 beta (IL-1β), and interferon gamma (IFN-γ). These lead to the up-regulation of neuronal damage effectors, including i-NOS (inducible nitric oxide synthase) expression and caspase 3 and 8 activation. Different free radicals, including NO, superoxide and hydrogen peroxide, cause lipid peroxidation and DNA damage to neurons and shut down mitochondrial respiration, leading to ultimately death [37]. Additionally, HLA-DR-expressed glial cells provide positive signals for the differentiation of CD4^+^ T helper cells into Th1 and Th17 subpopulations, which initiates a positive feedback loop in favor of neuronal damage by stimulating glial cells to secrete proinflammatory cytokines and suppress the secretion of IGF-1 (insulin-like growth factor), which acts as neuronal survival signal. HLA-DR-expressed glial cells also cause neuronal damage by acting on the Fas/FasL pathway [38]. However, BV PLA2 acts as a potent and novel negative regulator of these pathologic manifestations. It binds to lectin type CD206 receptors on dendritic cells (DC) and induces the expression of prostaglandin E2 (PGE2). In turn, PGE2 binds to EP2 receptors on naive Foxp3^-^ CD4^+^ cells and differentiates into Foxp3^+^CD4^+^T regulatory cells. These cells contribute to immune tolerance by shutting down the inflammatory manifestations specifically via microglial deactivation and decreased T cell infiltration [11].

Another life-changing neurodegenerative disorder is Alzheimer’s disease (AD). AD is caused by the formation of amyloid beta (Aβ) peptides in the central nervous system and is characterized by the development of extracellular senile plaques, intracellular neurofibrillary tangles and a reduction of neurons in the hippocampus and cerebral cortex. The clinical symptoms are dementia and impaired behavioral and cognitive function. BV PLA2 has an ameliorating effect on AD. To investigate this effect, Ye et al. [39] conducted an in vivo experiment using a 3xTg-AD mice model expressing three dementia-related transgenes; namely, APP_Swe_ (amyloid beta precursor protein), PS1_M146V_ (presenilin 1), and tau (P301 L; microtubule-associated protein tau). BV PLA2 was intraperitoneally administered at a dose of 1 mg/kg/per week from three months to six months of age in the experimental group. The test animals were subjected to a Morris water maze test to investigate their cognitive behavior. The BV PLA2-treated group showed improved cognitive function compared with the negative control group. The experimental animals were also subjected to brain glucose metabolism tests, cerebral histopathology, microglial activation patterns tests and CD4^+^T cells infiltration tests. The BV PLA2-treated group showed increased brain glucose metabolism, decreased deposition of Aβ peptide in the CA1 region of the hippocampus and decreased CD4^+^T cells. In addition, BV PLA2 had an ameliorating effect on the CD4^+^CD25+Foxp3+ Treg cell-mediated microglial inactivation.

**Figure 1:**
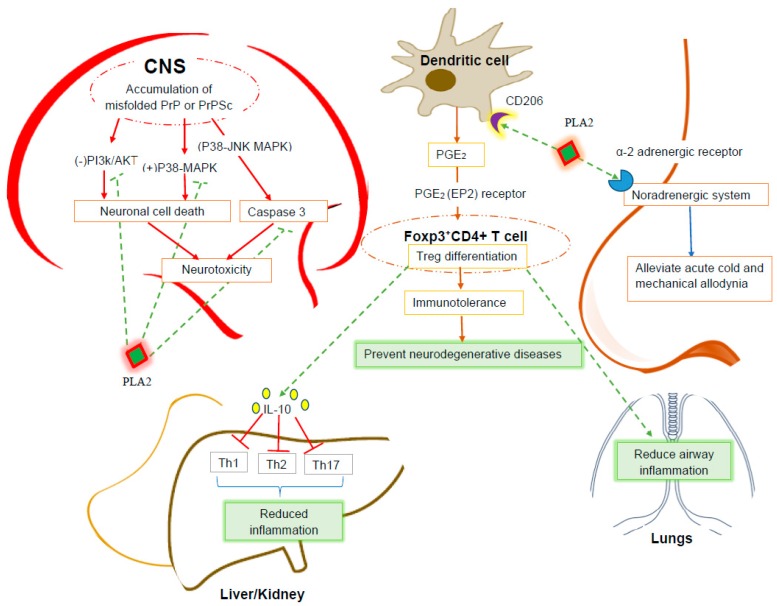
A schematic model of BV PLA2’s mechanism of actions in ameliorating neurodegenerative diseases, inflammatory diseases and asthma. Abbreviations: CNS: Central nervous system, PrP: Prion protein, PrPSc: Scrapie prion protein, JNK: Jun N-terminal kinases, p-38 MAPK: p38 mitogen-activated protein kinase, PI3k/AKT: Phosphatidylinositol-3-kinases/serine/threonine kinase, CD: Cluster of differentiation, PGE_2_: Prostaglandin E2, EP2: Prostaglandin E2 receptor, Foxp3+: Forkhead box P3, Treg: Regulatory T lymphocytes, IL-10: Interleukin-10, Th: T-helper cell, PLA2: Phospholipase A2.

BV PLA2 attenuates the neuronal cell death triggered by prion disease, a neurodegenerative disorder characterized by a proteinase K-resistant prion protein, (PrP) fragment (106–126). In prion disease, normal cellular prion protein (PrPC) undergoes conformational misconversion into scrapie prion protein (PrPSc). The accumulation of PrPSc triggers neuronal cell death in a mechanism associated with the blocking of the PI3K/AKT pathway and the activation of caspases and p38 mitogen-activated protein kinase (MAPK) pathways [40]. To investigate the neuroprotective effect of BV PLA2, Jeong et al. [18] conducted an ex vivo experiment with human neuroblastoma cell lines (SH-SY5Y). These cells were pre-treated with several different concentrations of BV PLA2 for one hour and were exposed to synthesized PrP (106–126). The cells were then subjected to an assay to determine the degree of PrP (106–126)-induced cell death. To reveal the protective mechanism of BV PLA2, the cells were also pre-treated with wortmannin (an AKT inhibitor) and SB-203580 (a p38 inhibitor). This study indicated that BV PLA2 counteracts the PrP (106–126)-induced cell death by blocking the activation of p38 mitogen-activated protein kinase (MAPK) pathways, the breakdown of caspases, and the attenuation of the PI3-AKT pathway. The overall effects of BV PLA2 on neurodegenerative diseases are summarized in Figure 1.

### 4.2. Anti-Inflammatory Effect of BV PLA2

Apitoxin has long been used to treat different inflammatory diseases, including lupus nephritis, cisplatin-induced nephrotoxicity, hepatotoxicity and allergic asthma (Figure 1).

BV PLA2 has a protective effect against acetaminophen-induced acute hepatotoxicity. To confirm this, Kim et al. [21] conducted an in vivo experiment with male C57BL/6 mice aged seven to eight weeks. The mice were intraperitoneally injected with BV PLA2 (0.2 mg/kg) once a day for five days before the induction of hepatotoxicity with a single dose administration of acetaminophen (500 mg/kg), a commonly used analgesic and anti-inflammatory drug. Following sacrifice, the liver ALT (alanine transaminase), AST (aspartate transaminase) enzyme profile and inflammatory cytokines IL-6, TNF and NO were assayed. The experimental group treated with BV PLA2 showed markedly decreased hepatotoxic parameters compared with the negative control group.

Kim et al. [41] also showed that BV PLA2 exerts a protective effect against cisplatin-induced nephrotoxicity by increasing the CD4+, CD25+, Foxp3+, Treg populations and IL-10 expression. In another study, Park et al. [14] showed that BV PLA2 ameliorates allergic airway inflammation. In this experiment, allergic airway inflammation was induced in male C57BL/6 mice (6–7 weeks of age) via intraperitoneal administration of ovalbumin (OVA) with aluminum hydroxide as an adjuvant. To investigate the protective effect of BV PLA2, differential bronchoalveolar lavage fluid (BLAF) cell count, histopathology and Th2 cytokine profiling were performed. The study found that BV PLA2 treatment causes decreased infiltration of neutrophils, eosinophils, lymphocytes, and macrophages in BLA fluid. It also attenuates periodic acid-Schiff (PAS)-positive goblet cell infiltration around the epithelia of the bronchial airway and down-regulates the expression of myosin regulatory light polypeptide 9 (MYL9) in the peribronchial muscle layer of the lungs compared with the negative control group. However, BV PLA2 treatment had no ameliorating effect on asthma in the CD206^−/−^ mice. Therefore, BV PLA2 protective effects are mediated by CD4^+^ CD25^+^ Foxp3^+^ Treg cellular function.

Radiotherapy is a very common anti-cancer treatment, but it is associated with severe side effects, such as radiation pneumonitis and late pulmonary fibrosis. These pathologic manifestations compromise the therapeutic value of radiation therapy and the quality of life of patients [42]. Dasomshin et al. [43] showed that BV PLA2 also has a CD4^+^CD25^+^Foxp3^+^ Treg cell-mediated protective effect against acute lung inflammation induced by radiotherapy. They irradiated C57BL/6 female mice (6 weeks old, weighing 20–25 g) with a single 75-Gy X-ray dose. After 3 weeks, they administered BV PLA2 (0.2 mg/kg) intraperitoneally on days 7, 10, 12, 14, 17 and 19 following irradiation. They found a marked decrease in inflammatory manifestations in the BV PLA2-treated experimental group compared with the negative control group. BV PLA2 decreased the infiltration of inflammatory cells in BLAF and down-regulated the gene expression of inflammasome-Nlrp1,3 (NLR family pyrin domain containing 1, 3) IL-1b, and Casp1 (caspase 1), chemokine-Mip1a (macrophage inflammatory protein 1a), Mcp1 (monocyte chemoattractant protein 1), and CCL4 (chemokine C-C motif ligand 4)), cytokine- (IL-6 and IL-17c) and fibrosis-related-Col3a1 (collagen type III alpha 1) and Fn1 (fibronectin 1). However, it had no effect on Treg depleted mice.

### 4.3. Anti-Nociceptive Effect of BV PLA2

In their experiment on a model of male C57BL/6 mice (6–8 weeks old), Li et al. [44] showed that BV PLA2 ameliorates oxilaplatin-induced side effects such as cold and mechanical allodynia. Oxilaplatin is used as a chemotherapeutic agent for metastatic colorectal cancer. In the mouse model, cold and allodynia were induced by administering oxilaplatin (6 mg/kg) intraperitoneally. The researchers confirmed that the administration of BV PLA2 (0.2 mg/kg) for five consecutive days attenuates allodynia. In addition, the mechanism of action of BV PLA2 was further investigated via the administration of *N*-(2-chloroethyl)-*N*-ethyl-2-bromobenzylamine hydrochloride (to deplete noradrenaline), dl-p-chlorophenylalanine (to deplete serotonin), idazoxan (α2-adrenegic receptor antagonist), prazosin (α1-adrenegic antagonist) along with BV PLA2 treatment. The administration of this regimen indicated that BV PLA2 exerts its anti-allodynic effect via α2-adrenergic receptors but not via the serotonergic system.

### 4.4. Anti-Cancer, Anti-Bacterial, Anti-Parasitic and Immunotherapeutic Effects of BV PLA2

In their early study, Putz et al. [45] showed the lytic and anti-proliferative actions of BV PLA2 and its substrate phosphatidylinositol-3,4-bis phosphate (PtdIns(3,4)P2) on different cancer cell lines, including the human kidney carcinoma cell line A498, the human breast carcinoma cell line T-47D, the human prostate carcinoma cell line DU145 and the human bronchial epithelium cell line BEAS-2B. Additionally, the tumor lysates produced stimulated the differentiation and maturation of monocyte-derived dendritic cells (moDC). The moDC cell populations can have an adjuvant role in immunotherapy against tumor cells. Putz et al. [46], in their specific study using the human kidney carcinoma cell line A498, endeavored to reveal the anti-tumorogenic mechanism of BV PLA2 and PtdIns (3,4)P2. They demonstrated that BV PLA2 produces lysophospholipids (lysophosphatidyl-choline, lyso-PC) by acting on membrane phospholipids. These form micelles and alter membrane organization and impair the proper function and expression of macromolecules and receptors (epidermal growth factor receptor) on the cell surface. Thus, the cell survival signal transduction pathway is disrupted at the first point [47]. These alterations also disrupt the signal transduction cascade mediated by PI3/Akt and extracellular signal regulated kinase (ERK1/2), which is required for cell survival. Lyso-PC also causes membrane damage by activating the Ca^2+^ channel and the protein kinase C-switching intracellular signal transduction pathway to generate oxygen-centered free radicals [48]. These cytotoxic actions greatly affect the rapidly proliferating tumor cells and kill them.

Several studies have also confirmed that BV PLA2 has significant anti-bacterial and antiparasitic activity. The in vitro study by Boutrin et al. [49] revealed that BV PLA2 has anti-protozoan activity against *Trypanosoma bruceibrucei* (i.e., the causative agent of African human trypanosomiasis), even at a low concentration (1 mg/mL), 30 min after administration by causing an imbalance and dysregulation of the calcium-mediated signal transduction pathway in parasitic cells. They also confirmed that BV PLA2 has anti-bacterial activity against *Enterobacter cloacae, Escherichia coli,* and *Citrobacter freundii* at minimum bactericidal concentrations of 0.01–0.001 µg/mL.

Different species of *Plasmodium* cause malaria. The parasites have to complete their development in the guts of specific types of mosquitos, which act as vectors. Luciano et al. [50] showed that the BV PLA2 gene construct (containing the promoter of *Anopheles gambiae* carboxypeptidase gene and 5‘ UTR and a signal peptide), which is expressed in the gut epithelium of *Anopheles stephensi,* negatively affects the development of *Plasmodium berghei* harbored within the gut of *A. stephensi* and impairs its transmission into naïve mice. The underlying mechanism of the anti-malarial effect of BV PLA2 is that it disrupts the interactions between *Plasmodium* and the intestinal cells of the mosquitoes’ mid-gut.

Moreover, in anticancer and antiviral immunotherapy, DCs (one type of professional antigen-presenting cell) play extraordinary roles by expressing antigens to both CD8^+^ cytotoxic T lymphocytes to kill malignant cells and CD4^+^ T helper cells to stimulate cytotoxic T cells. However, the internalization of candidate antigenic peptides in dendritic cells is very challenging. There are several approaches to this process, including the fusion of tumor cells with DCs [51]; incubation with cell lysates [52], apoptotic bodies [53], and heat shock protein-associated tumor proteins [54]; and tumor messenger RNA transfection [55] of viruses or large bacterial toxins to deliver antigenic DNA or proteins. However, these methods are not free of several adverse issues, such as the development of autoimmunity and cross-reactivity, competition between epitopes from the vector and the antigen for presentation, and safety and regulatory problems [56,57]. BV PLA2 (which is capable of irreversibly binding to the plasma membrane of any cell type via hydrophobic and electrostatic interactions with the anionic membrane phospholipids) can be exploited to anchor and internalize the candidate antigenic peptide fused to its C-terminal region. In their study, Almunia et al. reported that, to stimulate major histocompatibility complex (MHC) class I peptide cross-presentation and MHC class II peptide presentation for the preparation of cell-based vaccines, BV group III sPLA2 histidine-34 is replaced with glutamine (BV group III sPLA2H34Q) devoid of catalytic activity and is effective as a membrane-binding vector [58].

### 4.5. Biochemical and Physiological Roles of Hyaluronidase

The hyaluronidase (Hya) in BV acts as spreading factor for venom by degrading hyaluronan (a constituent of the extracellular matrix) in the skin. It shows more than a 50% sequence homology with hymenoptera and as much as 30% homology with some mammalian hyaluronidases. It plays a significant role in the development of allergic manifestations. A clinical study showed that approximately 78% of patients who are allergic to BV have recombinant Hya-specific IgE antibodies [59]. As a ubiquitous enzyme, Hya is expressed in a wide range of organisms and human tissues. Hya in higher vertebrates is associated with a wide range of physiological and pathological processes, such as fertilization, wound healing, embryonic development, angiogenesis, diffusion of drugs and toxins, metastatic mechanisms, inflammatory manifestations and meningitis [60]. Nevertheless, the full potentiality of BV hyaluronidase has not been explored.

### 4.6. Physiological Role of BV Acid Phosphatase

BV acid phosphatase is another major allergen. It contains several IgE-binding epitopes. The acid phosphatase-IgE interactions stimulate the basophils to degranulate and trigger the onset of a hypersensitivity type I reaction, which is characterized by the formation of wheals and urticaria and, in more severe cases, anaphylactic shock [61,62].

## 5. Future Approaches

BV PLA2 is the main component and allergen of BV, and it orchestrates the functions of different inflammatory cells, including mast cells, neutrophils, microglia, and CD4 + Th2 cells. Elen et al. showed that BV PLA2 administered via sub-plantar injection at a dose of 5–30 µg/paw can induce the rapid onset of edema in mice [13]. Although BV PLA2 has a significant anti-neurodegenerative effect, it also exerts a neurotoxic effect that is mediated by its binding to N-type receptors, which are highly expressed in brain cells. Nicolas et al. showed that the binding of BV PLA2 is mediated by the interactions of the interfacial binding surface, the hydrophobic channel and the Ca^2+^ binding loop domain [63]. Liu et al. showed that upon the administration of BV PLA2 into the thoracic spinal cord of rats, extensive demyelination occurs [64]. The administration of sPLA2 into the cervical dorsolateral funiculus of rats causes demyelination, neuroglial cell loss and axonopathy in a dose-dependent manner [65]. Therefore, it is necessary to take careful provisions, not only with BV PLA2 but with all BV enzymes, to avoid these adverse effects, and the optimal doses and treatment methods required to avoid adverse effects should be determined in further experiments, including preclinical and clinical studies.

## 6. Conclusions

Previous studies have advanced our knowledge about BV, and the information assembled in this review will serve as an updated resource regarding BV enzymes. The evidence compiled in this review indicates that BV enzymes have a number of pharmacotherapeutic effects on the human body via miscellaneous mechanistic pathways. Further studies are warranted to elucidate the detailed physiochemical properties and exact pharmacotherapeutics and mechanisms of BV enzymes and enzyme-like proteins, especially phospholipase B, hyaluronidase, acid phosphatase and α-glucosidase.

## Figures and Tables

**Table 1:** Identification methods and biological functions of recognized BV enzymes.

BV Enzyme	Identification Method	Biological Function	Reference
PLA2	Reversed phase HPLC on different columns with an acetonitrile-water-trifluoro acetic acid eluent system, nano-LC/MALDI-TOF/TOF-MS system, shotgun LC-FT-ICR MS analysis	Causes destruction of phospholipids and dissolves the cell membrane of blood cells. Lowers blood coagulation and blood pressure, prevents neuronal cell death caused by prion peptides. Prevents acetaminophen-induced hepatotoxicity through CD4^+^, CD25^+^, Foxp3^+^ and T cells (Treg) in mice.	[17,18,19,20,21]
Phospholipase B	Paper chromatography and also paper electrophoresis	Causes cleavage of lysolectin and thus detoxification. Has the combined activity of PLA1 and PLA2. Enhances the activity of PLA2. Cleaves lysophospholipid into glycerophosphocholine and anionic fatty acids.	[16,18,19,20,22]
Hyaluronidase	Reverse-phase HPLC on different columns with an acetonitrile-water-trifluoroacetic acid eluent system, nano-LC/MALDI-TOF/TOF-MS system, shotgun LC-FT-ICR MS analysis	Degrades hyaluronic acid, enabling the penetration of venom into tissue. Causes dilation and increased permeability of blood vessels and thus increased blood circulation.	[17,18,19,20]
Acid phosphatase	1-D SDS PAGE in gel digest followed by LC-ESI-LTQ-FT-ICR-MS, nano-LC/MALDI-TOF/TOF-MS system, shotgun LC-FT-ICR MS analysis	Potent histamine releaser from human sensitized basophils. May be exploited in immunotherapy against BV.	[23]
α-glucosidase	Salting-out chromatography, DEAE-cellulose, DEAE-sepharose CL-6B, Bio-Gel P-150, and CM-toyopearl 650M column chromatographies, shotgun LC-FT-ICR MS analysis	Involved in honey production.	[24,25]

Abbreviations: HPLC: High-performance liquid chromatography; DEAE cellulose: Diethylaminoethyl cellulose; 1D-SDS PAGE: 1-dimensional sodium dodesylsulphate gel electrophoresis; LC-FT-ICR MS analysis: Liquid chromatography Fourier transform ion cyclotron resonance mass spectrometry analysis; MALDI: Matrix-assisted laser desorption ionization.

**Table 2:** Investigated information regarding *Apis mellifera* BV enzymes.

	Phospholipase A2				Hyaluronidase				Acid Phosphatase				α-Glucosidase			
**Nomenclature and Source**																
Recommended name	Phospholipase A_2_				Hyaluronoglucosaminidase				Venom acid phosphatase				Alpha glucosidase			
Alternative names	Allergen Api m I, Allergen: Api m 1, lecithinase A, phosphatidase, phosphatidolipase, phospholipase A				Allergen Api m II, Allergen: Api m 2, hyaluronoglucosidase, chondroitinase, chondroitinase I, BVH, HAse, HyaI, Mu toxin, sperm surface protein PH-20				Allergen: Api m 3, acid phosphatase				Acid maltase, AGL, α-1,4-glucosidase, α-d-glucosidase, α-glucopyranosidase, α-glucoside hydrolase, α-glucosidase III, glucoinvertase, glucosidoinvertase, glucosidosucrase, maltase, maltase glucoamylase, HBG III, HBGase I, HBGase II, HBGase III			
Systematic name	Phosphatidylcholine 2-acylhydrolase				Hyaluronate 4-glycanohydrolase				Phosphate-monoester phosphohydrolase (acid optimum)				α-d-glucosideglucohydrolase			
Short name	bvPLA2				Hya				Acph-1				AGL			
Popular sources	Mouse, human, bovine, rat, fruit fly				Human, zebrafish, rat, bovine				Rice, *Arabidopsis thaliana,* fruit fly, human, *Caenorhabditis elegan*, bees, viper snakes				Fruit fly, human, mouse, *Saccharomyces cerevisiae*, *Arabidopsis thaliana*			
**Organism-based information**																
Source tissue	Venom gland				Expressed in the venom glands of worker bees; it is also detected in the testes of drones but not in the queen’s BV gland or in pupae				Venom gland				Hypopharyngeal gland of worker bees			
Subcellular location	Extracellular				Extracellular				Extracellular				No data			
Maximal levels	Approximately 40 micrograms PLA2/venom sac				No data				No data				No data			
Percentage of dry weight (%)	10–12				1–2				1				0.6			
Purification	Dialysis, column chromatography Native enzyme of BV, as confirmed by gel filtration to homogeneity				Ion exchange chromatography at pH 5.0; HiTrap HP SP column chromatography Recombinant His-tagged enzyme				A combination of saturated ammonium sulfate precipitation, gel filtration and ion exchange chromatography				Homogeneity via salting-out chromatography and five other chromatographic steps			
**Enzymatics**																
EC number	3.1.1.4				3.2.1.35				3.1.3.2				3.2.1.20			
Family	Phospholipase A2 family; group III subfamily				Glycosyl hydrolase 56 family				Venom acid phosphatase				Glycoside hydrolase family 13			
Reaction type	Hydrolysis of carboxylic ester				Hydrolysis of O-glycosyl bond				Acid phosphatase activity				Hydrolysis			
Reaction	Phosphatidylcholine + H_2_O = 1-acylglycerophosphocholine + a carboxylate				Random hydrolysis of (1→4)-linkages between *N*-acetyl-beta-d-glucosamine and-glucuronate residues in hyaluronate				Phosphatidylcholine + H_2_O = 1-acylglycerophosphocholine + a carboxylate				Hydrolysis of terminal, non-reducing (1→4)-linked α-d-glucose residues with release of α-d-glucose			
Metabolic pathways	Alpha-linolenic acid metabolism Linoleic acid metabolism Arachidonic acid metabolism Lipid metabolism Ether lipid metabolism Glycerophospholipid metabolism Biosynthesis of secondary metabolites Aspirin-triggered resolving D biosynthesis Aspirin-triggered resolving E biosynthesis Phosphatidylcholine acyl editing Phospholipases Plasmalogen degradation				Glycosaminoglycan degradation				Aminobenzoate degradation Microbial metabolism in diverse environments NAD metabolism NAD phosphorylation and dephosphorylation NAD salvage pathway III NAD/NADH phosphorylation and dephosphorylation Phosphate acquisition Vitamin B1 metabolism				Galactose metabolism Glycogen degradation I Glycogen metabolism Starch sucrose metabolism Starch degradation I Starch metabolism			
Metals and ions	Ca^2+^, Others: Ba^2+^, Mg^2+^, Sr^2+^				No data				No data				Ca^2+^			
Inhibitors	1-hexadecyl-3-trifluroethylglycero-sn-2-phosphoethanol AnMIP EDTA Maleic anhydride Manoalogue Omega-bromo-4-nitroacetophenone				Beta 1,4-galacto-oligosaccharides Glutathione *N*-acetyl-l-cysteine Partially sulfated neomycin Partially sulfated planteose Partially sulfated verbascose Sulfated hydroquinone galactoside				Not found				1-deoxynojirimycin 2,6-anhydro-1-deoxy-1-[(1-oxopentyl-5-hydroxy)amino]-d-glycero-d-ido-heptitol 2,6-anhydro-7-deoxy-7-([1-(hydroxymethyl)ethenyl]amino)-d-glycero-l-gulo-heptitol 2,6-anhydro-7-deoxy-7-[(1-methylethenyl)- amino]-d-glycero-l-gulo-heptitol 2,6-anhydro-7-deoxy-7-[(1-phenylethenyl) amino]-d-glycero-l-gulo-heptitol 2,6-anhydro-7-deoxy-7-[(3-hydroxy-1-methylidenepropyl)amino]-d-glycero-l-gulo-heptitol 2,6-anhydro-7-deoxy-7-[(4-hydroxy-1-methylidenebutyl)amino]-d-glycero-l-gulo-heptitol 2,6-anhydro-7-deoxy-7-[(6-hydroxy-1-methylidenehexyl)amino]-d-glycero-l-gulo-heptitol Acarbose Castanospermine Conduritol B epoxide D-gluconolactone Deoxynojirimycin Dithiothreitol Miglitol Voglibose			
Activators	Acylating agents EDTA				Not investigated				Not investigated				Not investigated			
Active sites	Histidine active site Aspartic active site				Not investigated				Histidine active site				Eight-stranded alpha/beta barrel			
Optimum pH	7.4–8				3.6 and/or 3.8				4.8				5.5			
Optimum temperature (°C)	25				37 and/or 62				45				No data			
Temperature stability ((°C)	20				4–90 (34% activity at temperatures as low as 4 °C; still exhibits hydrolase activity at 90 °C at 19% of the optimum level) 90 (19% activity at 90)				No data				40 (stable up to) 60 (15 min, complete inactivation)			
**Genomics**																
Gene symbol	PLA2				LOC406146				Acph-1				Hbg3			
Other names	bvpla2, GB13351				GB18543				GB12546				GB19017			
Gene type	Protein coding				Protein coding				Protein coding				Protein coding			
RefSeq status	Provisional				Provisional				Validated				Provisional			
Location	Chromosome LG13				Chromosome LG14				Chromosome LG5				Chromosome LG6			
Exon count	4				8				12				14			
**Proteomics**																
Formula	C_833_H_1284_N_234_O_252_S_14_				C_1990_H_3087_N_547_O_567_S_16_				C_2082_H_3199_N_519_O_596_S_11_				C_2979_H_4445_N_759_O_873_S_22_			
Total number of atoms	2617				6207				6407				9078			
Molecular weight (Dalton)	19,057.6				44,259.6				45,389.0				65,564.9			
Length	167				382				388				567			
Theoretical pI	7.05				8.67				5.63				5.06			
Total number of negatively charged residues (Asp + Glu)	21				46				51				75			
Total number of positively charged residues (Arg + Lys)	21				50				44				56			
Estimated half-life	30 h (mammalian reticulocytes, in vitro) >20 h (yeast in vivo) >10 h (*Escherichia coli*, in vivo)				30 h (mammalian reticulocytes, in vitro) >20 h (yeast in vivo) >10 h (*Escherichia coli*, in vivo)				30 h (mammalian reticulocytes, in vitro) >20 h (yeast in vivo) >10 h (*Escherichia coli*, in vivo)				30 h (mammalian reticulocytes, in vitro) >20 h (yeast, in vivo) >10 h (*Escherichia coli*, in vivo)			
Instability index	27.60 (this classifies the protein as stable)				44.30 (this classifies the protein as unstable)				53.41 (this classifies the protein as unstable)				32.19 (this classifies the protein as stable)			
Grand average hydropathicity (GRAVY)	65.93				−0.455				−0.326				−0.457			
Graphical overview	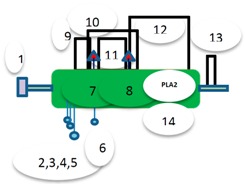				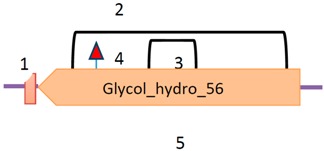				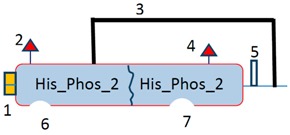				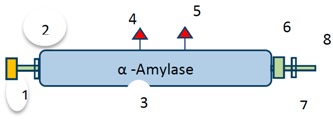			
	**Source**	**Domain**	**Start**	**End**	**Source**	**Domain**	**Start**	**End**	**Source**	**Domain**	**Start**	**End**	**Source**	**Domain**	**Start**	**End**
	1 = Sig P	n/a	1	18	1 = Transmembrane	n/a	12	30	1 = Sig P	n/a	1	15	1 = Sig P	n/a	1	17
	14 = pfam	Phospholipid A2-2	35	132	5 = pfam	Glyco hydro 56	38	367	6 = pfam	His_Phos_2	16	187	2 = Low complexity	n/a	44	56
	Low complexity	n/a	4	15	Low complexity	His_Phos_2	15	26	7 = pfam	His_Phos_2	162	315	3 = pfam	α-Amylase	49	394
					Disorder	n/a	231	236	Low complexity	n/a	233	244	6 = disorder	n/a	400	410
													7 = disorder	n/a	412	414
													8 = Low complexity	n/a	424	433
	**Disulfide**				**Disulfide**											
	9 = Disulfide, coordinates 42–64				2 = Disulfide, coordinates 54-345											
	10 = Disulfide, coordinates 63–103				3 = Disulfide, coordinates 221-233											
	11 = Disulfide, coordinates 70–96															
	12 = Disulfide, coordinates 94–128															
	13 = Disulfide, coordinates 138–146															
	**Annotation**				**Annotation**				**Annotation**				**Annotation**			
	7 = H-active site, position 67				4 = E-active site: proton donor, position 145				2 = H-active site nucleophile, position 26				4 = D-active site nucleophile, position 223			
	8 = D-active site, position 97								4 = E-active site: proton donor, position 273				5 = E-active site: proton donor, position 286			
